# Supplementary material for: Identification of photoperiod-induced specific miRNAs in the adrenal glands of Sunite sheep (Ovis aries)
Source: Front Vet Sci. 2022 Jul 22;9:888207. doi: 10.3389/fvets.2022.888207 (PMC9354845; doi:10.3389/fvets.2022.888207)
Supplement: Supplementary file 1 [file Data_Sheet_1.ZIP › Supplementary file/miRDeep2 file.zip!/miRDeep2 file/Know_Structure/LP42A1/oar-let-7a.pdf]

miRBase precursor : oar-let-7a
Total read count : 367544
oar-let-7a read count : 365511
remaining reads : 2033

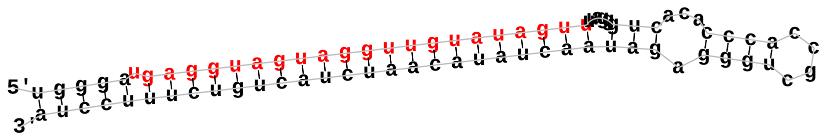

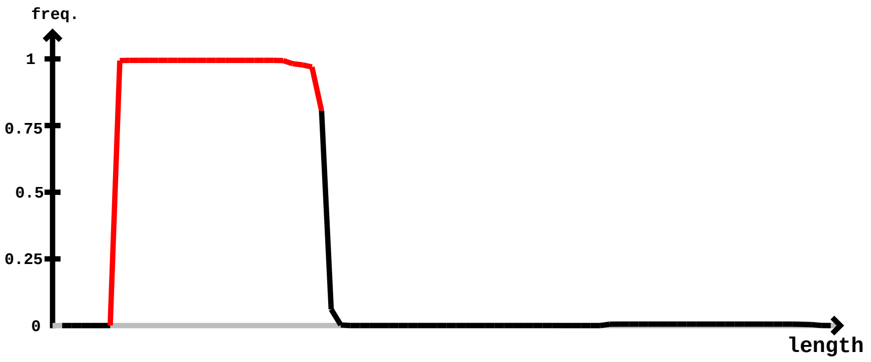

oar-let-7a

```
5'- ugggaugagguaguagguuguauaguuuuagggucacacccaccgcugggagauaacuauacaaucuacugucuuuccua   -3'    exp
    (((((.(((((((((((((((((((((.....(((...((((....)))).)))))))))))))))))))))))))))))    reads      mm         sample
    ...gUugagguaguagguuguauaguu.....................................................    2          1          seq
    ....augagguaguagguugua..........................................................    1          0          seq
    ....augagguaguagguuguau.........................................................    1          0          seq
    ....Uugagguaguagguuguaua........................................................    1          1          seq
    ....augagguaguagguuguauagu......................................................    2          0          seq
    ....Uugagguaguagguuguauagu......................................................    4          1          seq
    ....Uugagguaguagguuguauaguu.....................................................    10         1          seq
    ....augagguaguagguuguauaguu.....................................................    20         0          seq
    ....augagguaguagguuguauaguuu....................................................    3          0          seq
    .....ugagguagGagguugua..........................................................    31         1          seq
    .....ugagguaguaAguugua..........................................................    7          1          seq
    .....ugagguaguUgguugua..........................................................    2          1          seq
    .....ugagguaguagguugua..........................................................    277        0          seq
    .....ugaggCaguagguugua..........................................................    1          1          seq
    .....ugagguaguaAguuguau.........................................................    25         1          seq
    .....ugagguaguagguuUuau.........................................................    6          1          seq
    .....ugaAguaguagguuguau.........................................................    44         1          seq
    .....ugagguaguagguuguau.........................................................    3846       0          seq
    .....ugagguUguagguuguau.........................................................    2          1          seq
    .....ugagguagGagguuguau.........................................................    381        1          seq
    .....ugagguaguagguuguaua........................................................    1480       0          seq
    .....ugagguagGagguuguaua........................................................    148        1          seq
    .....ugagguagGagguuguauag.......................................................    352        1          seq
    .....ugagguaguagguuguauag.......................................................    2549       0          seq
    .....ugagguaguagguuguauagu......................................................    53025      0          seq
    .....ugagguagGagguuguauagu......................................................    7196       1          seq
    .....ugagguaguagguuguauaguu.....................................................    251488     0          seq
    .....ugagguagGagguuguauaguu.....................................................    22012      1          seq
    .....ugagguaguagguuguauaguuu....................................................    21580      0          seq
    .....ugagguaguagguuguauaguuuA...................................................    121        1          seq
    .....ugagguaguagguuguauaguuuu...................................................    656        0          seq
    .....ugagguaguagguuguauaguuuua..................................................    8          0          seq
    ......gagguaguagguuguau.........................................................    1          0          seq
    ......gagguaguagguuguaua........................................................    1          0          seq
    ......gagguagGagguuguauag.......................................................    1          1          seq
```

oar-let-7a

```
ugggaugagguaguagguuguauaguuuuagggucacacccaccgcugggagauaacuauacaaucuacugucuuuccua
......gagguaguagguuguauag.......................................................    1     0    seq
......gagguagGagguuguauagu......................................................    4     1    seq
......gagguaguagguuguauagu......................................................    27    0    seq
......gagguaguagguuguauaguu.....................................................    156   0    seq
......gagguagGagguuguauaguu.....................................................    10    1    seq
......gagguaguagguuguauaguuu....................................................    20    0    seq
......gagguaguagguuguauaguuuu...................................................    1     0    seq
.......agguaguagguuguauagu......................................................    2     0    seq
.......agguaguagguuguauaguu.....................................................    1     0    seq
.......agguaguagguuguauaguuu....................................................    1     0    seq
........gguaguagguuguauaguu.....................................................    1     0    seq
..........uaguagguuguauaguu.....................................................    1     0    seq
..........uaguagguuguauaguuuuag.................................................    1     0    seq
..........uaguagguuguauaguuuuagg................................................    1     0    seq
.............uagguuguauaguuuuaggguc.............................................    2     0    seq
...........................uuagggucacacccaccgcugg...............................    5     0    seq
...........................uuagggucacacccaccgcuggg..............................    2     0    seq
...........................uuagggucacacccaccgcuggga.............................    19    0    seq
...........................uuagggucacacccaccgcugggagau..........................    1     0    seq
....................................................auaacuauacaaucuacuguc.......    1     0    seq
.....................................................uaacuauacaaucuacugucu......    1     0    seq
.....................................................uaacuauacaaucuacugucuuu....    5     0    seq
.....................................................uaacuauacaaucuacugucuuuc...    1     0    seq
......................................................aacuauacaaucuacugucuuu....    1     0    seq
........................................................cuauacaaucuacuguc.......    1     0    seq
........................................................cuauacaaucuacugucu......    3     0    seq
........................................................Auauacaaucuacugucuu.....    1     1    seq
........................................................cuauacaaucuacugucuu.....    4     0    seq
........................................................cuauacaaucuacuguUuuu....    1     1    seq
........................................................cuauacaaucuacugucuuu....    302   0    seq
........................................................cuauacaaucuacugucuuuc...    324   0    seq
........................................................cuauacaaucuacugucuuucc..    1206  0    seq
........................................................cuauacaaucuacugucuuuccu.    58    0    seq
.........................................................uauacaaucuacugucuuu....    1     0    seq
.........................................................uauacaaucuacugucuuucc..    13    0    seq
.........................................................uauacaaucuacugucuuuccu.    61    0    seq
.........................................................uauacaaucuacugucuuuccua    1     0    seq
..........................................................auacaaucuacugucuuucc..    1     0    seq
..........................................................auacaaucuacugucuuuccu.    15    0    seq
..........................................................auacaaucuacugucuuuccua    3     0    seq
```
